# Personality Disorders and Development

**DOI:** 10.3390/brainsci12080983

**Published:** 2022-07-26

**Authors:** Eva Möhler

**Affiliations:** Department of Child and Adolescent Psychiatry, Saarland University Hospital, G-66421 Homburg, Germany; eva.moehler@uks.eu

Personality disorders constitute a major challenge for society, as well as psychiatry and psychotherapy. Specifically, in connection with emotionally unstable personality traits, large and rigorous studies [1] emphasize the high costs for health and other services services.

Recent research has featured different levels of personality functioning, that can be measured early in development and result in specific profiles for different types of personality disorders [2]. Even for children as young as school age, diagnostic tools have recently been developed These novel tools are able to help understand the onset and timing in the development of personality disorders, e.g., a recent study could demonstrate that especially emotion dysregulation (ED) contributes to self-injurious behavior in a large group of adolescents presenting to a child and adolescent psychiatric emergency service [3]. Several authors also mention emotionally dysregulated behavior as one of the leading symptoms of Borderline Personality disorder [4].

The occurrence of typical phenomena associated with personality dysfunction in early development, such as severe tantrums, low frustration tolerance, aggression, negative mood and suicidality is even higher with estimates of about 45% in psychiatric patients [5]. 30% of adults with emotionally unstable personality report having injured themselves as early as at primary school age [6]. Therefore a focus on developmental aspects of personality disorders seems to be of crucial importance for elucidating underlying factors and mechanisms.

On the level of the brain, emotional instability has been reported to be correlated to reduced glucose metabolism in premotoric areas, in the dorsolateral prefrontal cortex, in parts of the anterior prefrontal cortex, in the thalamus, in the caudate nucleus and in the lenticular nucleus [7] and in the bilateral medial orbitofrontal cortex [8]. CSF tests in emotionally unstable patients and other personality disorders showed particularly low levels of 5-hydroxyidol acetic, the degradation product of serotonin. A negative correlation between the amount of 5-hydroxyidol acetic acid and the frequency of suicidal behaviour could be established [9].

In addition, a significantly higher serotonin transporter binding was observed [10]. From these observations, a serotonergic dysfunction can be concluded as a partial aspect of emotional instability [11]. As further correlates on the neurobiological level, correlations between a reduction of serotonergic activity in the brain and subsequent impulsive aggressiveness, as well as between an increase in cholinergic system reactivity and affective instability have been described [12]. The neuroanatomical correlation to serotonergic dysfunctioning is still unclear. A reduced serotonergic activity in the frontal and cingulate cortex in combination with noradrenergic dysfunction is postulated to lead to an increase in emotional responsiveness of the amygdala and enhances dopaminergic projection onto orbitofrontal and corticolimbic structures via mutual connection to the ventral tegmental area. This disturbed alternating circuit might be associated with impulsive and emotionally dysregulated behavior [9].

On the neuroanatomical level, the orbitofrontal cortex seems to play a special role in emotion regulation. The orbitofrontal cortex takes over the inhibition of aggression with the help of the anterior cingulate cortex. Furthermore, the orbitofrontal cortex also shows a regulatory influence on the amygdala by inhibition [13]. Patients with lesions in the orbitofrontal cortex were reported to show a significantly higher degree of impulsivity and a higher frequency of inadequate behaviour compared to patients in the healthy control group accordingly [9]. Oxytocin has also been postulated to be a major component affecting social behavior and perception in personality disorders [13]. The neurobiological abnormalities found so far are indirectly associated with personality factors such as neuroticism, impulsiveness, anxiety, affective instability and uncertain attachment behaviour in conjunction with different expression of these factors via dominance or inactivation of the various neurotransmitter systems for an effect on the psychological situation or the possible development of a borderline personality disorder [14].

On another important note, research on personality disorders has stressed the importance of trauma and abusive experiences, as well as adverse childhood experiences for the development of personality disorders. In general, neurobiology as well as physiology has been described to be vastly altered in victims of childhood abuse, e.g., [15]. Generally, a considerable number of studies have pointed out the important role of childhood adversities (CA) such as physical and sexual abuse, neglect, parental loss or family conflict in the development of psychopathology in adulthood, emphasizing the need to consider ACEs as an influential factor in models examining personality development. Literature repeatedly points out that especially the experience of trauma, especially in the form of sexual abuse and physical abuse, but also emotional neglect in childhood or adolescence plays a major role in the development of personality disorder [16]. It can be assumed that a longer-term experience of stress or abuse during the unfinished development of regulatory and coping mechanisms leads to restrictions of adequate effect control and behavioural control. In several studies up to 85% of patients with emotional instability reported childhood abuse, around 70% of patients with an emotional unstable personality disorder report physical abuse and 67% report sexual abuse. Furthermore, in a study of Bohus [17], a large number of patients are massively psychosocially burdened by sexual violence (65%), physical violence (60%) or neglect (40%). The retrospective evaluation of patient histories and data from a structured clinical interview for DSM-IV (SKID) confirmed stressful early life experiences as possible etiological factors. A large proportion of patients examined reported traumatic experiences such as sexual and physical violence in childhood and/or adulthood. In addition, patients with previous experience of sexual violence had an increased number of suicide attempts compared to the group of patients without such experiences in the past.

With regard to predictors on the level of relationships, in families with known personality disorders, problems with attachment behaviour between children and their parents are described significantly more frequently in retrospect [18]. According to the basic assumptions of attachment theory experience with interpersonal interactions form the basis for the development of emotional health, disturbed attachment behaviour leads to interactional difficulties and disorders with self-control in the child’s development [19]. Dramatic relationships with disorganized, chaotic, conflictual and sensitive family interactions, a chaotic and hostile family atmosphere, as well as an invalidating style in upbringing, in which feelings, thoughts and behaviour are neither taken seriously nor accepted, play a special role [20]. With a disabling, disrespectful and hardly supportive or predictable environment, traumatic experiences have a stronger impact on the development of a personality in childhood [13]. Particularly early occurrence of abuse, separation or neglect has been described to be closely related to the development of uncertainties in attachment behavior [18]. The available data show that a substantial body of research associates early life conditions with later personality disorder, however, to date very few studies have been conducted prospectively or with regard to targets for treatment or prevention.

Therefore, strategies regarding prevention and intervention such as dialectic behavioral therapy tend to focus on and target different levels of personality functioning, such as self-control, empathy and interpersonal behavior. Much progress has been made in this regard, e.g., with mentalization based therapies and other novel treatment approaches. Specifically, regarding the patients’ frequent mistrust and relationship difficulties, low threshold programs need to be developed and evaluated.

In summary, research and clinical provider need better equipment with diagnostic and psychotherapeutic tools for characterization of personality disorders and a much more thorough understanding of the developmental pathways through which early experience shapes personality. In addition, a more dimensional framework respecting the normal variation of human feelings and behavior and regarding it as highly susceptible to environment and therefore therapeutic interventions is encouraged [21]. Novel insights on these aspects can help clinicians to tailor intervention efforts more precisely [22].

Therefore, studies focusing on pathogenetic aspects of personality disorders by addressing neurobiological underpinnings and childhood adversity are collected in this issue. Furthermore, interventions and therapies that give an overview on established therapeutic tools such as DBT and the younger ‘derivatives’ are of interest, as well as articles describing novel interventions developed from the recent ED-Framework. Together with review articles on state of the art advancements in personality disorders, research in this issue also explores the adverse childhood experiences framework and aims to describe empirical research on neurobiological associations between trauma and personality disorders.

With original articles and reviews on pathogenesis, diagnosis, treatment and classification of personality disorders our aim is to achieve with this issue a large transdiagnostic long-term benefit for research as well as clinical aspects. This is of crucial significance, since understanding and improving personality functioning in all aspects prevents the onset of multiple associated psychiatric and physical disorders and severe psychsoscial dysfunction. Intervention and preventive efforts to promote successful social and professional development will be shown and highly welcomed in this issue. The global alliance for prevention and early intervention of borderline personality disorder has named the development and radnomized controlled evaluation as a novel public health priority [23], which should be validated not only, but also by this special issue.
